# Endoscopic Management of Diverticular Bleeding

**DOI:** 10.1155/2014/353508

**Published:** 2014-12-09

**Authors:** Tarun Rustagi, Thomas R. McCarty

**Affiliations:** ^1^Section of Digestive Diseases, Department of Internal Medicine, Yale University School of Medicine, 333 Cedar Street, 1080 LMP, New Haven, CT 06520, USA; ^2^Department of Internal Medicine, Yale University School of Medicine, New Haven, CT 06520, USA

## Abstract

Diverticular hemorrhage is the most common reason for lower gastrointestinal bleeding (LGIB) with substantial cost of hospitalization and a median length of hospital stay of 3 days. Bleeding usually is self-limited in 70–80% of cases but early rebleeding is not an uncommon problem that can be reduced with proper endoscopic therapies. Colonoscopy is recommended as first-line diagnostic and therapeutic approach. In the vast majority of patients diverticular hemorrhage can be readily managed by interventional endotherapy including injection, heat cautery, clip placement, and ligation to achieve endoscopic hemostasis. This review will serve to highlight the various interventions available to endoscopists with specific emphasis on superior modalities in the endoscopic management of diverticular bleeding.

## 1. Introduction

Lower gastrointestinal bleeding (LGIB) is a common reason for hospitalization in the United States with an annual incidence of 36 per 100,000 [1]. Of the various causes of LGIB, diverticular hemorrhage remains the most common source [1, 2]. Colonic diverticular bleeding is commonly described as brisk hematochezia accounting for 30–50% of cases of massive rectal bleeding [3–5]. Diverticular hemorrhage accounts for approximately 20–48% of LGIB with a median length of hospital stay of 3 days [6–10].

The pathophysiology of diverticular bleeding involves thinning of the mucosa most commonly along the fundus or neck of the diverticula with associated injury to the penetrating vessel. When these diverticula develop near intramural branches that supply the colon, repeated microtrauma leads to eccentric thickening and thinning of the media and results in segmental weakness of the vasa recta predisposing to hemorrhage [11]. During colonoscopy, active bleeding is more commonly seen in the base and nonbleeding visible vessel in the neck [12, 13]. While diverticular disease occurs predominately in the left colon in the Western population, diverticula in the right colon are more likely to bleed [14–16]. Several theories are suggested for the high prevalence of right colonic sources of diverticular bleeding including a larger diameter of right colon diverticula and thinner mucosal walls [16].

## 2. Associated Risks of Bleeding

Bleeding usually is self-limited in 70–80% of cases but recurs in 22–38% after the first episode and more than 50% after the second episode [17]. Old age, smoking, alcohol consumption, use of nonsteroidal anti-inflammatory medications (NSAIDs), aspirin, antithrombic drugs, and presence of bilateral diverticulosis and atherosclerosis related diseases (hypertension, diabetes mellitus, ischemic heart disease, and obesity) have been suggested as risk factors for colonic diverticular hemorrhage and also to increase the risk of long-term recurrent bleeding [18–23]. Risks of early rebleeding (defined as clinical evidence or visualization on endoscopy within 30 days after initial treatment), which is most likely to contribute to prolonged hospitalization, include signs of shock and active bleeding seen on colonoscopy [24].

## 3. Bowel Preparation and Identification

With such a high percentage of spontaneous hemorrhage resolution, 83% of urgent colonoscopies (defined as <24 hours) are negative for any overt bleeding or stigmata of recent hemorrhage (SRH) [25]. SRH is defined by active bleeding from a diverticulum, a nonbleeding visible vessel, or an adherent clot. This low yield can be improved with aggressive preparation prior to colonoscopy; however, no randomized controlled trials exist to compare 4-liter versus 2-liter preparations [26]. Despite improved results with aggressive preparation measures, achieving excellent or even fair bowel preparation for urgent colonoscopy to identify subtle diverticular bleeding is varied and is a difficult goal to achieve. In a retrospective study of 110 patients with colonic diverticular bleeding by Mizuki et al., preparation with polyethylene glycol preparation compared to no preparation allowed for a higher rate of identifiable bleeding diverticula (28.2% versus 12.0%, *P* = 0.11) though not statistically significant [27]. Additionally, the detection rate was significantly higher when colonoscopic examination was performed within 18 hours of the final hematochezia than when it was performed after 18 hours (40.5% versus 10.5%, *P* < 0.01). Even with the delay required prior to endoscopy and the difficulty in achieving adequate preparation, preparation is an important and necessary aspect of colonoscopy. Additional use of a water-jet scope to remove debris has been described to increase SRH detection with colonoscopy, though it warrants further evaluation [28].

Apart from logistic difficulty in coordinating the procedure after-hours, disadvantages of urgent colonoscopy include the risks of sedation, invasive nature, and rare but serious complications of perforation [29]. However, complication rate is only modestly increased for urgent colonoscopy compared with elective colonoscopy in comparison trials though no head to head trial has been attempted to date (0.6% versus 0.3%, resp.) [30]. Many of these limitations and disadvantages might also apply to other diagnostic modalities such as angiography and radionucleotide scanning which may be able to localize the bleeding site. Green et al. randomized patients presenting with LGIB to urgent colonoscopy (within 8 hours) or a standard care algorithm (red blood cell scan in patients with suspected active bleeding while those without active bleeding underwent elective colonoscopy within 1–4 days after presentation) [31]. Patients with a positive red blood cell scan went to visceral angiography (with treatment if actively bleeding) while those with a negative scan had an elective colonoscopy. A definite source of bleeding was identified in 42% of patients in the urgent colonoscopy group, compared to only 22% in the standard care group [31]. Of the patients undergoing urgent colonoscopy, more than half of the preparations were graded fair to poor, thereby underscoring the importance of adequate bowel prep prior to endoscopy. Overall, this randomized controlled trial demonstrated colonoscopy to be a superior diagnostic test. Despite the study being underpowered for other major outcomes, the use of tagged red blood cell scan and angiography should be limited to cases where the bleeding site cannot be identified with colonoscopy or endoscopic therapeutic attempts are unsuccessful. Overall, urgent colonoscopy, as both a means of definitive diagnosis and therapeutic intervention, remains advantageous to identify SRH, provides a multitude of therapeutic options for appropriate treatment, and, while being controversial, may reduce the need for blood transfusion, length of hospital stay, and risk of rebleeding [26, 31].

## 4. Timing of Colonoscopy

Although limited by small sample size and statistical power, early studies showed no benefit in clinical outcomes with the use of urgent colonoscopy (within 12–48 hours) compared to routine or delayed colonoscopy in the management of LGIB [29, 31, 32]. As mentioned above, Green et al. showed no significant difference in clinical measures comparing urgent colonoscopy to tagged red blood cell scan and elective colonoscopy [31]. However, a recent large study using the 2010 Nationwide Inpatient Sample (NIS) dataset of 58,296 discharges of LGIB (12,746 (21.9%) of which were diverticular hemorrhage) found early colonoscopy (performed within 24 hours) to be associated with statistically significant outcomes [6]. While there was no difference in mortality in patients with LGIB who underwent early versus delayed colonoscopy (0.3% versus 0.4%, *P* = 0.24), early colonoscopy was associated with a shorter length of hospital stay (2.9 versus 4.6 days, *P* < 0.001), decreased need for blood transfusion (44.6% versus 53.8%, *P* < 0.001), and lowered overall hospitalization costs ($22,142 versus $28,789, *P* < 0.001) [6]. On multivariate analysis, timing of colonoscopy did not affect mortality (adjusted odds ratio of 1.5; 95% CI, 0.7–2.7) and delayed colonoscopy was associated with an increase in the length of hospital stay by 1.6 days and an increase in hospitalization costs of $7,187 [6].

## 5. Endoscopic Treatment Options

Current American Society for Gastrointestinal Endoscopy (ASGE) guidelines recommend “early” colonoscopy for management of LGIB [33]. Given the increased risk of early rebleeding associated with the presence of active bleeding seen during colonoscopy, identification of SRH allows for appropriate endoscopic intervention including injection, heat cautery, clip placement, and ligation [26, 34, 35]. With many options to achieve endoscopic hemostasis it is first important to determine the appropriate method. If visualization of diverticulum with the SRH is obscured by blood, water-jet scope irrigation and suctioning and/or injection of dilute epinephrine for initial control of active bleeding may be of benefit [12]. Intravenous glucagon can also be administered to control peristalsis and achieve better visualization.

## 6. Epinephrine Injection and Electrocautery

As discussed above, one option for achieving endoscopic hemostasis includes four-quadrant submucosal injection of dilute epinephrine (1 : 10000). Cessation of bleeding from an active diverticulum using this technique was first described in 1985 [36]. This form of treatment, however, often provides only temporary cessation of hemorrhage with significant risk of early rebleeding within 30 days [35]. Therefore, epinephrine injection monotherapy is not preferred and should be combined with another endoscopic treatment modality to achieve more durable results (combination or dual endoscopic therapy, similar to upper GI ulcer bleeding). Although multipolar electrocautery is commonly used in treatment of upper GI ulcer bleeding, its use in management of diverticular bleeding is limited by the inherent risk of full-thickness injury with high risk of perforation and is particularly not suitable for treatment of lesions not visible at the surface of the diverticulum [37, 38]. Bloomfeld et al. demonstrated a higher rate of rebleeding with bipolar coagulation and much more common use of epinephrine injection needed to control hemorrhage in those without signs of rebleeding [35]. Injection and heat probe cautery also are not amenable to everted diverticulum [26]. With the aforementioned shortcomings, submuscosal injection and electrocautery should not be used as first line endoscopic monotherapy for diverticular bleeding.

## 7. Endoscopic Hemostatic Clipping

Hokama et al. were the first to report the successful treatment of colonic diverticular bleeding using hemostatic clips with no immediate or recurrent bleeding in a small series of 3 patients [39]. The endoclips are placed adjacent to the visible site of bleeding and then closed allowing for occlusion of vessel to achieve hemostasis. Hemostatic clip placement theoretically offers the advantage of less injury to the mucosa and adjacent tissues compared to coagulation therapy [39–41]. Direct clipping of the exposed vessel or erosions is superior to clipping of the entire diverticular orifice (reefing method) in patients with no active bleeding [42]. These clips typically fall off after some time, theoretically after hemostasis has been obtained and cessation of bleeding is achieved. However, given the pathophysiology of diverticular bleeding, thinning of the mucosa and poor segmental integrity of the vessel may lead to ineffective long term hemostasis by simple clipping. As discussed earlier, right colon is the most common location for diverticular hemorrhage. Separate studies by Kominami et al. and Ishii et al. have shown active hemorrhage and location in the ascending colon to be significant predictors of refractory colonic diverticular bleeding after endoscopic clipping [42, 43]. In another study, endoclip treatment preceded by epinephrine injection achieved 100% reduction in early bleeding; however, during a median follow-up of 15 months, late recurrent bleeding occurred in 18.2% of patients [40]. Additionally, a retrospective study by Kaltenbach et al. found recurrent bleeding occurred in about 21% of patients treated with clipping at 43 months [44]. A further study found that treatment with endoclip closure in a zipper fashion may be suboptimal for occlusion of the immediate source of bleeding and the underlying artery in question [45]. Given this significant risk of long-term rebleeding, indirect placement of hemoclips in ascending lesions is ineffective and should not be considered first line for endoscopic treatment of diverticular bleeding, especially if right-sided disease is suspected.

## 8. Endoscopic Band Ligation

Endoscopic band ligation (EBL) also achieves immediate hemostasis with fewer complications and is even an option in massive bleeding [34, 46]. The procedure involves the placement of the transparent sleeve or hood of the banding device adjacent to the suspected diverticulum of interest. With application of a single-band ligator over the diverticulum and eversion with minimal suction, the band can be deployed to ligate the everted diverticulum. Farrell et al. demonstrated the effectiveness of this endoscopic approach on both surgically removed specimens (ex vivo) and in patients with actively bleeding colonic diverticula [47]. This study showed no rebleeding or need for surgery in any of the 4 patients during a follow-up period of 12 months. While this study was small, it showed EBL to be a safe and effective alternative for treatment of persistent or recurrent diverticular bleeding. A distinct advantage of EBL for definitive diverticular hemorrhage is the ability to suction compliant tissue and eversion for better visualization of SRH in the base of bleeding diverticula [12]. In a study of 29 patients with 31 diverticula with SRH (17 of which were right-sided lesions), early rebleeding (defined as clinical evidence of recurrent LGIB within 30 days after initial treatment) after EBL occurred in 11% of patients with no procedure-related complications [34].

EBL may serve as a permanent follow-up therapy in patients with recurrent bleeding who fail prior endoscopic attempts. Ishii et al. reported a patient who developed sudden massive hematochezia and hypotension 2 days after initial endotherapy with placement of 3 endoclips for diverticular bleeding [48]. The rebleeding was treated with EBL with no clinical evidence of further bleeding or any complications at the 4-month follow-up. In a study of 66 patients (18 receiving EBL, 48 receiving endoclips), EBL was superior to hemoclips in reduction of early rebleeding [45]. EBL and endoscopic clipping both showed an initial success rate of 100% with no complications; however, early rebleeding was significantly lower in the EBL-treated group (6% versus 33%, *P* = 0.018). Unfortunately, this study did not subsequently treat patients with endoclip failures with EBL to evaluate for improvement in bleeding. Nonetheless, EBL is considered superior to clipping in the treatment of colonic diverticular hemorrhage and should be attempted as the initial therapy, especially for right-sided disease.

## 9. Conclusion

In summary, urgent colonoscopy is an appropriate and recommended first line strategy to identify a diverticular cause of acute LGIB. Despite the disadvantages of colon preparation and low prevalence of SRH, colonoscopy provides the ability to identify bleeding source regardless of bleeding rate or presence of bleeding and provides multiple therapeutic possibilities of treatment with EBL emerging as a preferred modality of intervention. Early identification of diverticular hemorrhage by colonoscopy is associated with shorter length of hospital stay, decreased use of blood transfusion, and overall lower hospitalization costs.
